# Novel loss of function mutation in *TUBA1A* gene compromises tubulin stability and proteostasis causing spastic paraplegia and ataxia

**DOI:** 10.3389/fncel.2023.1162363

**Published:** 2023-06-23

**Authors:** Riccardo Zocchi, Emanuele Bellacchio, Michela Piccione, Raffaella Scardigli, Valentina D’Oria, Stefania Petrini, Kristin Baranano, Enrico Bertini, Antonella Sferra

**Affiliations:** ^1^Unit of Neuromuscular Disorders, Translational Pediatrics and Clinical Genetics, Bambino Gesù Children’s Hospital, IRCCS, Rome, Italy; ^2^Molecular Genetics and Functional Genomics Research Unit, Bambino Gesù Children’s Hospital, IRCCS, Rome, Italy; ^3^Research Laboratories, Bambino Gesù Children’s Hospital, IRCSS, Rome, Italy; ^4^Consiglio Nazionale delle Ricerche (CNR), Institute of Translational Pharmacology (IFT), Rome, Italy; ^5^European Brain Research Institute (EBRI) “Rita Levi-Montalcini,” Rome, Italy; ^6^Department of Neurology, The Johns Hopkins University School of Medicine, Baltimore, MD, United States

**Keywords:** tubulin, microtubule, tubulinopathies, TUBA1A, mutation, gene, neurodegeneration

## Abstract

Microtubules are dynamic cytoskeletal structures involved in several cellular functions, such as intracellular trafficking, cell division and motility. More than other cell types, neurons rely on the proper functioning of microtubules to conduct their activities and achieve complex morphologies. Pathogenic variants in genes encoding for α and β-tubulins, the structural subunits of microtubules, give rise to a wide class of neurological disorders collectively known as “tubulinopathies” and mainly involving a wide and overlapping range of brain malformations resulting from defective neuronal proliferation, migration, differentiation and axon guidance. Although tubulin mutations have been classically linked to neurodevelopmental defects, growing evidence demonstrates that perturbations of tubulin functions and activities may also drive neurodegeneration. In this study, we causally link the previously unreported missense mutation p.I384N in TUBA1A, one of the neuron-specific α-tubulin isotype I, to a neurodegenerative disorder characterized by progressive spastic paraplegia and ataxia. We demonstrate that, in contrast to the p.R402H substitution, which is one of the most recurrent TUBA1A pathogenic variants associated to lissencephaly, the present mutation impairs TUBA1A stability, reducing the abundance of TUBA1A available in the cell and preventing its incorporation into microtubules. We also show that the isoleucine at position 384 is an amino acid residue, which is critical for α-tubulin stability, since the introduction of the p.I384N substitution in three different tubulin paralogs reduces their protein level and assembly into microtubules, increasing their propensity to aggregation. Moreover, we demonstrate that the inhibition of the proteasome degradative systems increases the protein levels of TUBA1A mutant, promoting the formation of tubulin aggregates that, as their size increases, coalesce into inclusions that precipitate within the insoluble cellular fraction. Overall, our data describe a novel pathogenic effect of p.I384N mutation that differs from the previously described substitutions in *TUBA1A*, and expand both phenotypic and mutational spectrum related to this gene.

## 1. Introduction

Tubulins are the structural subunits of microtubules, highly dynamic cytoskeletal structures involved in a wide variety of cellular functions, such as directed vesicle transport, cell motility and polarity (Bouchet and Akhmanova, 2017; Garcin and Straube, 2019; Meiring et al., 2020). They play a key role in neuronal cells, supporting their architecture and allowing the long-range motor driven transport within the axon (Holzbaur and Scherer, 2011; Yogev et al., 2016). Microtubules are also involved in neuronal migration by coordinating nucleokinesis and providing the tractional force to extend the leading process, and are critical for the establishment and the maintenance of neuronal polarity (van Beuningen and Hoogenraad, 2016; Lasser et al., 2018; Thyagarajan et al., 2022).

All eukaryotes express multiple, distinct genes of α and β-tubulins. Humans have nine isoforms of both α and β-tubulin, which possess specific tissue and developmental distributions (Roll-Mecak, 2020). To date at least 8 tubulin genes (*TUBB3*, *TUBA1A*, *TUBB2A*, *TUBB2B*, *TUBB*, *TUBA4A*, *TUBA4B*, *TUBG1*) have been reported to be expressed in brain (Miller et al., 1987; Gloster et al., 1994, 1999; Leandro-García et al., 2010; Breuss et al., 2012, 2015; Hersheson et al., 2013; Rustici et al., 2013; Latremoliere et al., 2018; Ivanova et al., 2019). Mutations in these isotypes have been mainly linked to non-progressive cortical and sub-cortical development malformations collectively known as “tubulinopathies” and including lissencephaly, polymicrogyria, cortical dysplasia and microcephaly (Breuss et al., 2012; Poirier et al., 2013; Cushion et al., 2014; Sferra et al., 2020). Nevertheless, mutations in *TUBB3*, *TUBA4A*, *TUBB4A*, and *TUBB2A* have been recently associated to neurodegenerative phenotypes, thus demonstrating that mutations in tubulin genes can cause also neurodegeneration (Lohmann et al., 2013; Tischfield et al., 2013; Smith et al., 2014; Curiel et al., 2017; Sferra et al., 2018; Watanabe et al., 2018).

The class 1 α-tubulin (TUBA1A) is the most highly expressed α-tubulin in developing neurons and provides over 95% of the α-tubulin in the embryonic brain (Lewis et al., 1985; Miller et al., 1987; Gloster et al., 1994, 1999; Zhang et al., 2014). Moreover, it has been demonstrated that this isoform constitutes a consistent percentage of the total α-tubulins in cortical and spinal neurons over time, thus playing a critical role in adult neuronal microtubule network as well (Buscaglia et al., 2020).

Perturbations of TUBA1A cause a wide spectrum of lissencephaly and brain malformations which are often accompanied by motor impairment, epilepsy and cognitive deficits (Bahi-Buisson et al., 2014; Myers et al., 2015; Yokoi et al., 2015; Hebebrand et al., 2019; Xie et al., 2021; Schröter et al., 2022). Although the p.N102D missense heterozygous mutation in Tuba1a has been associated with adult-onset ataxia in mice, no similar degenerative phenotype has been recognized in humans (Buscaglia et al., 2020, 2022).

Here we report on the identification of a novel variant in *TUBA1A*, identified by WES in a subject with ataxia and progressive spastic paraplegia. We show that, differently from the previously reported *TUBA1A* substitutions linked to lissencephaly, and analogously to the heterozygous p.N102D mutation associated to adult-onset ataxia in mice, the present disease-causing variant affects TUBA1A stability and proteostasis, promoting its aggregation and preventing its incorporation into microtubules. We have also proved that the affected residue is critical for the proper tubulin stability, since the pathogenic effect of I384N substitution was independent of the other tubulin isotypes. Lastly, we demonstrate that the inhibition of the proteasome system increases the protein levels of TUBA1A^*I*384*N*^, promoting the formation of TUBA1A aggregates that, as their size increases, coalesce and precipitate into the detergent-insoluble protein fraction, in a faster mode compared to TUBA1A*^WT^*.

In conclusion, the present study expands the phenotypic spectrum of *TUBA1A* beyond structural brain defects, and describes a novel pathogenic mechanism associated to a novel loss of function mutation in *TUBA1A* human gene, suggesting that the reduced availability of this α-tubulin, due to its increased protein instability and proteostasis, may be enough to induce neurodegeneration in human.

## 2. Materials and methods

### 2.1. Whole exome sequencing

NGS sequencing was performed by a trio WES and 100% of the coding region of *TUBA1A* was covered at a minimum of 10X by the XomeDx Test.

### 2.2. Cell culture and transfection

Human embryonic kidney 293 (HEK-293) and COS-1 cells were cultured in Dulbecco’s modified Eagle’s medium (Invitrogen, Thermo Fisher Scientific) supplemented with 10% fetal bovine serum (Gibco), 1% Sodium Pyruvate (Invitrogen, Thermo Fisher Scientific) and 1% penicillin/streptomycin solution (Invitrogen, Thermo Fisher Scientific), in a 5% CO_2_ atmosphere at 37°C. COS-1 cells were seeded in 6-well plates on sterile, untreated glass coverslips for confocal microscopic analysis. HEK-293 and COS-1 cells were seeded in 6-well plates for all experiments of immunoblot analysis. A total of 24 h after being seeded, HEK-293 and COS-1 cells were transfected with 1 μg of expression vector per well, using Lipofectamine 2000 (Invitrogen, Thermo Fisher Scientific) according to the manufacturer’s instructions.

D7-E2_GFP neural progenitors (Medelin et al., 2018; Rossi et al., 2020) were cultured in Dulbecco’s modified Eagle’s medium/F12 medium (Invitrogen, Thermo Fisher Scientific) supplemented with B27 (Thermo Fisher Scientific), epidermal growth factor and basic fibroblast growth factor (20 and 10 ng/ml, respectively; Peprotech) in a 5% CO_2_ atmosphere at 37°C. Growth factors were replenished weekly. For transfection experiments, D7-E2 GFP neural progenitor cells were seeded in 6-well plates at 80% confluence and 24 h after being seeded, transfected with 4 μg of expression vector per well, using Lipofectamine 2000 (Invitrogen, Thermo Fisher Scientific) according to the manufacturer’s instructions.

### 2.3. Plasmid constructs

Wilde type (WT) FLAG-tagged TUBA1A and TUBA1B expression vectors were generated by polymerase chain reaction amplification of reverse-transcribed RNA isolated from human primary fibroblasts, using a specific primer annealing at the 5′ region with an artificial *Not*I site overlapping the start codon and a specific primer annealing at the 3′ regions overlapping the stop codon and containing an artificial site *Bam*HI. After *Not*I and *Bam*HI digestion, the full length coding regions were cloned into pFLAG vector (Invitrogen, Thermo Fisher Scientific) and the single-nucleotide changes resulting in the p.I384N or in the p.N102D substitutions were introduced by site-directed mutagenesis (Stratagene). The entire transgene-coding regions were sequence validated following maxiprep yields (Qiagen).

WT FLAG-tagged TUBA4A expression vector was generated by polymerase chain reaction amplification of reverse-transcribed RNA isolated from human primary fibroblasts, using a specific primer annealing at the 5′ region with an artificial *Not*I site overlapping the start codon and a specific primer annealing at the 3′ region overlapping the stop codon and containing an artificial site *Eco*RV.

After *Not*I and *Eco*RV digestion, the full length coding region was cloned into pFLAG vector (Invitrogen, Thermo Fisher Scientific) and the single-nucleotide change resulting in the p.I384N substitution was introduced by site-directed mutagenesis (Stratagene). The entire transgene-coding regions were sequence validated following maxiprep yields (Qiagen).

### 2.4. MG132 treatment

A total of 24 h after transfection, HEK-293 and COS-1 cells over-expressing WT and mutated FLAG-tagged TUBA1A recombinant proteins were treated with 20 μM MG132 (Sigma-Aldrich) or with the same volumes of DMSO (untreated), at 37°C in a 5% CO_2_ atmosphere, for 4, 8, and 16 h. At these defined times, HEK-293 cells were washed twice with PBS and collected for protein extraction, while COS-1 cells were fixed and processed for immunofluorescence.

### 2.5. Immunofluorescence analysis

In total, 24 h after transfection and 4, 8, 16 h after MG132 treatment, cells were fixed with ice–cold methanol for 7 min. Cells were then blocked with 5% bovine serum albumin for 1 h and subsequently incubated in a humidified chamber with the primary mouse anti-FLAG antibody (1:500, Sigma-Aldrich, 2 h). After primary incubation, the cells were washed three times with PBS and incubated for 1 h with a secondary goat anti-mouse antibody conjugated to AlexaFluor 555 (1:500, Molecular Probes, Invitrogen), protected from light. Cells were then washed three times with PBS and incubated with Hoechst (1:10.000, Merck, Germany) to counterstain nuclei. Primary and secondary antibodies were diluted in 1% bovine serum albumin, Hoechst was diluted in PBS. Coverslips were mounted using 50% glycerol/PBS and analyzed by Confocal microscopy. Confocal microscopy was performed on an Olympus FV3000laser-scanning confocal microscope (Evident Scientific, Hamburg, Germany) equipped with 405 nm and 561 nm laser sources. Sequential confocal images were acquired using a UPLXAPO 40x oil immersion objective (1.40 numerical aperture) with a 1024 × 1024 format, scan speed 8 μs/pixel and z-step size of 0.4 μm. Maximal Intensity Projection (MIP) of each Z-reconstruction was obtained by FV31S-SW (version 2.4.1.198) Olympus software.

### 2.6. Protein extraction and immunoblot analysis

D7-E2_GFP neural progenitors were collected 48 h after transfection, COS-1 cells were collected 24 h after transfection and HEK-293 cell were collected 24 h after transfection and 4, 8, 16 h after MG132 administration. Cells were then washed twice with PBS and lysed on ice with Radioimmunoprecipitation assay buffer (50 mM Tris–HCl, pH 7.4, 50 mM NaCl, 0.1% Nonidet P-40, 1% Triton, 0.5% sodium deoxycholate) supplemented with phosphatase and protease inhibitors (Pierce, Thermo Fisher Scientific) for 20 min. Protein extracts were centrifugated at 12,000 × *g* at 4°C for 15 min and the supernatants were collected and placed in separate tubes.

For HEK-293 cells treated with MG132, the cell pellet, obtained after centrifugation and corresponding to the detergent insoluble fraction, was resuspended with Laemmli buffer, using the same volume of the supernatant, corresponding instead to the detergent soluble fraction. Cell pellets were then boiled for 20 min.

Protein concentrations were assessed using a BCA assay (Invitrogen, Thermo Fisher Scientific). A total of 30 μg of soluble fraction and an equal volume of insoluble fraction were loaded and run on a 4–12% mini protein gel (Invitrogen, Thermo Fisher Scientific). Samples were transferred to a polyvinylidene fluoride membrane (Immobilon, Merck) at 30 V ON at 4°C. Immediately following transfer, the membrane was rinsed in TBS-tween, blocked with 5% milk diluted in TBS for 1 h, and then immunoblotted with the following primary antibodies: FLAG (1:2000, Sigma-Aldrich), β-actin (1:2000, Abcam), GAPDH (1:10.000, Sigma-Aldrich).

### 2.7. Microtubule partioning into soluble and polymerized fractions

A total of 24 h after transfection, HEK-293 cells were gently washed with pre-warmed PBS and resuspended in 100 μl of tubulin extraction buffer (Boggs and Cabral, 1987) containing 20 mM Tris–HC1, pH 6.8, 0.14 M NaC1, 0.5% Nonidet P-40, 1 mM MgC12, 2 mM EGTA, 4 ug/ml paclitaxel (Sigma-Aldrich) supplemented with phosphatase and protease inhibitors (Pierce, Thermo Fisher Scientific) at room temperature. The microcentrifuge tubes containing 100 μl of lysed cell contents were briefly mixed (Vortex Genie mixer, setting 10) and then centrifuged at 12,000 × *g* for 10 min at 4°C. The supernatant, containing the soluble tubulin, was carefully separated from the pellet, containing the polymerized tubulin, and placed in separate tubes. Pellet were resuspended in 100 μl of Laemmli buffer and boiled for 40 min.

### 2.8. Quantitative real-time PCR

Total mRNA from transiently transfected HEK-293 cells was isolated using Trizol Reagent (Sigma-Aldrich), according to the manufacturer’s protocol. One microgram of each RNA samples was reverse transcribed with the SuperScript First-Strand Synthesis system and random hexamers as primers (LifeTechnologies). The expression levels of *TUBA1A* mRNA were measured by quantitative real-time PCR (qRT-PCR) in an ABI PRISM 7500 Sequence Detection System (LifeTechnologies) using Power SYBR Green I dye chemistry. For the qRT-PCR experiments we used a Forward primer annealing in the FLAG region of *TUBA1A* construct and a Reverse primer annealing in the exon 2 of *TUBA1A.* Data were analyzed using the 2-Delta-Delta Ct method with hypoxanthine guanine phosphoribosyltransferase (HPRT) as housekeeping gene and data are shown as fold change relative to control. Notably, the use of an alternative housekeeping gene such as the TATA box binding protein (*TBP*) gene did not modify the data obtained with *HPRT* gene.

### 2.9. Data analysis and statistics

Statistical analysis was performed using the GRAPHPAD/Prism 7.0 Software (San Diego, CA, USA). The Mann-Whitney test was used for comparisons between two groups. Comparisons of more than two groups were performed using the one-way ANOVA for parametric data or using the Kruskal-Wallis test and analyzed *post-hoc* using the Dunn’s test, for non-parametric data. All data are presented as mean ± standard error of the mean (SEM). Statistical analyses used in each experiment are indicated in their respective figure legends.

### 2.10. Homology modeling of the human tubulin alpha-1A chain

Molecular drawings were made with PyMOL^1^.

## 3. Results

### 3.1. Clinical assessment

**FIGURE 1:**
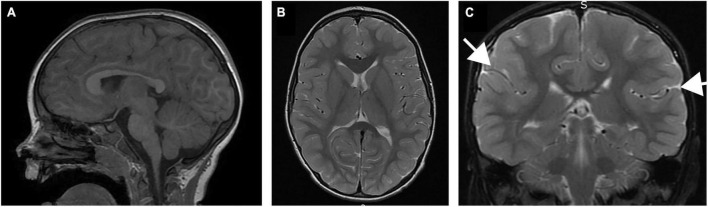
Brain MRI of patient performed at 22 months of age. **(A)** Sagittal T1 image demonstrating normal size and morphology of the corpus callosum, cerebellum and brainstem. **(B)** Coronal T2 image demonstrating normal configuration of the basal ganglia, including fiber organization of the internal capsule. **(C)** Coronal T2 image demonstrating subtle thickening in the sylvian fissure (arrows).

The patient was initially evaluated at 35 months of age. He was the third product of healthy parents and he was born to a 35 year old mother and father. The pregnancy was naturally conceived and the gestation was unremarkable. He was born at term, weighing 8 lbs, 15 oz. There were no neonatal concerns, and he has generally been a medically healthy youngster, with no chronic medical problems and his only surgery was for PE tube placement for chronic otitis media. The family was initially concerned about motor delays at 10-11 months of age. He walked independently at the age of 2 years. At this time, he was noted to have lower extremity spasticity, and his gait was described as ataxic, without any evidence of other cerebellar signs such as nystagmus or dysmetria. He was followed by ophthalmology starting at 6 months of age for concern for poor tracking, and subsequently strabismus and ptosis were noted to develop with time. Initial brain imaging was performed at 22 months of age, which was read as normal, but second opinion review subsequently appreciated subtle focal thickening of the posterior sylvian cortex with associated decreased white matter. There was no evidence of abnormalities of the corpus callosum or disorganization of the fibers of the internal capsule in the region of the basal ganglia, and the cerebellum was normal (Figure 1). Subsequent imaging confirmed a normal spine MRI without evidence of cord tethering.

The patient was last evaluated at 6 years of age documenting that the disease had progressed. His head circumference was 50.5 cm (20th percentile). There were no dysmorphic features of note but marked ptosis was present, and he had worsened from the first evaluation. He had marked difficulty with pursuits; there was no nystagmus. There was no spasticity in the uppers, but he had marked spasticity in the lower extremities with brisk reflexes at the patellas, clonus at the Achilles, and upgoing toes bilaterally. He had a spastic gait with truncal ataxia, but no evidence of dysmetria or tremor. Subsequently, he was diagnosed with congenital fibrosis of the extraocular muscles and underwent strabismus surgery at 6.5 years of age. He underwent selective dorsal rhizotomy for progressive lower extremity spasticity at 6.5 years with good results. Formal neuropsychological testing at nearly age 7 revealed a full scale IQ of 89 and a diagnosis of ADHD.

### 3.2. Molecular analysis

To identify the molecular event underlying the trait, exome sequencing was performed in the proband and unaffected parents.

Clinical trio whole exome sequencing revealed the pathogenic *de novo* variant c.1151T > A (p.I384N) in heterozygous state in *TUBA1A* gene (NM_006009.2) (ClinVar, Accession:VCV000585140.1).

Whole exome sequencing was performed in a clinical laboratory (GeneDx, Gaithersburg, MD, USA). Genomic DNA was extracted from a blood sample. The DNA was enriched for the complete coding regions and splice site junctions for most of the human genome using a proprietary capture system developed by GeneDx for next-generation sequencing. The enriched targets were simultaneously sequenced with paired-end reads on a Illumina platform. Bi-directional sequence reads were assembled and aligned to reference sequences based on NCBI RefSeq transcripts and human genome build GRCh37/USC hg19. Using a custom-developed analysis tool (XomeAnalyzer), data were filtered and analyzed to identify sequence variants.

The p.I384N variant was not observed in large population cohorts (Lek et al., 2016) and *in silico* analyses, including protein predictors and evolutionary conservation, supported the hypothesis that TUBA1A is intolerant to this variation.

### 3.3. Structural analysis on the effects of the I384N variant of TUBA1A

**FIGURE 2:**
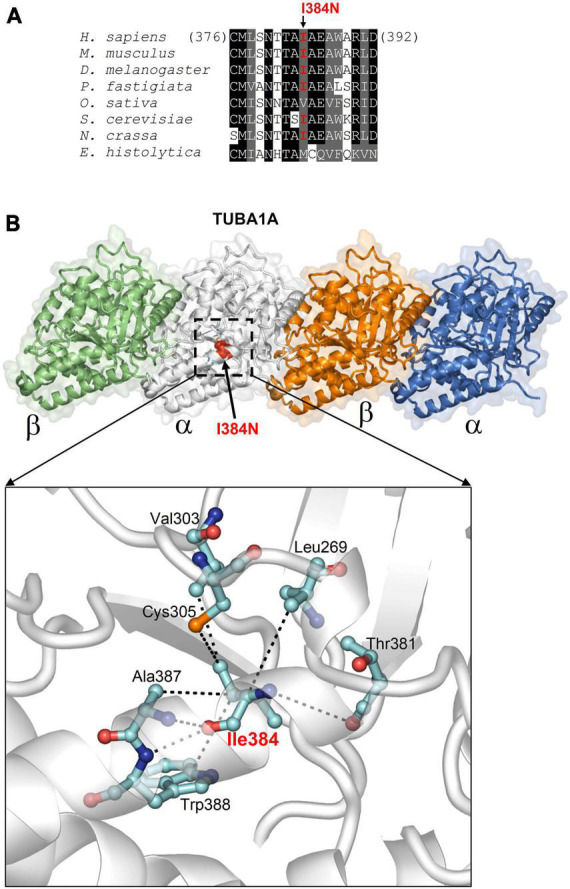
*In silico* structural analysis and alignment of the p.I384N amino acid change. **(A)** Protein sequence alignment across species around the human tubulin alpha-1A chain residue affected by the I384N replacement (columns with identical and similar residues are in black and gray background). In organisms very distant from the human, substitutions only occur with other aliphatic residues with hydrophobicity similar to isoleucine. **(B)** Structure of tubulin alpha-1A chain heterodimerized with the β- chain (from the Protein Data Bank entry 7Z6S; the tubulin chains are shown in different colors) and position of Ile384 (indicated in red in one a chain). The enlarged view highlights how Ile384, facing the interior side of the protein, employs both side chain and backbone atoms in multiple hydrophobic and polar interactions (dotted lines) to determine the specific fold of the site [reproduced with permission from “GeneDx”(1/31/23)].

The p.I384N mutation affects an isoleucine residue highly conserved among TUBA1A orthologs and engaged in several interactions critical for the structure of α-tubulin-1A chain (Figure 2). These interactions are both hydrophobic and polar as they involve the side chain, and also the backbone nitrogen and oxygen atoms of isoleucine in position 384 (Figure 2), and are predicted to be impaired by the Ile to Asn replacement, since it introduces a hydrophilic amino acid. Thus, the Ile384Asn change is predicted to destabilize the TUBA1A structure resulting in the misfolding of the tubulin chain.

### 3.4. The p.I384N substitution disrupts TUBA1A stability and inhibits its assembly into microtubule network

**FIGURE 3:**
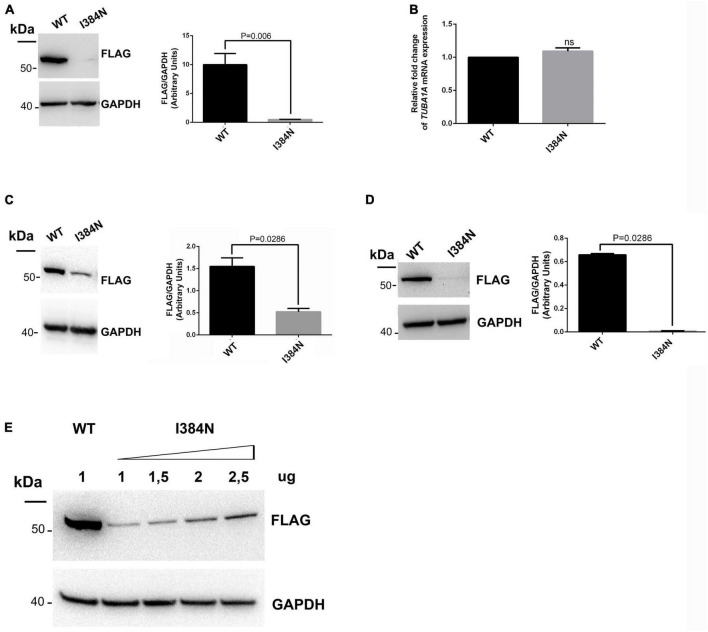
The p.I384N substitution affects TUBA1A protein stability. **(A)** Immunoblot analysis of HEK-293 cells transiently transfected with FLAG-tagged TUBA1A (WT and mutant) documented a significant reduction of mutant tubulin. GAPDH was used as loading control. Histogram shows the quantitative analysis for western blot. The graph reports the mean ± SEM. Data were analyzed by Mann Whitney test *N* = 7. **(B)** Gene expression of TUBA1A in transiently transfected HEK-293 cells detected by qRT-PCR did not documented differences in *TUBA1A* mRNA levels. Expression values were normalized to *HPRT* expression. Histogram shows the quantitative analysis of *TUBA1A* gene expression. The graph reports the mean ± SEM. Data were analyzed by Mann Whitney test *N* = 3. **(C)** Immunoblot analysis of COS-1 cells transiently transfected with FLAG-tagged TUBA1A (WT and mutant) documented a significant reduction of mutant tubulin. GAPDH was used as loading control. Histogram shows the quantitative analysis for western blot. The graph reports the mean ± SEM. Data were analyzed by Mann Whitney test *N* = 4. **(D)** Immunoblot analysis of D7-E2_GFP neural progenitors transiently transfected with FLAG-tagged TUBA1A (WT and mutant) documented a significant reduction of mutant tubulin. GAPDH was used as loading control. Histogram shows the quantitative analysis for western blot. The graph reports the mean ± SEM. Data were analyzed by Mann Whitney test *N* = 4. **(E)** Immunoblot analysis of HEK-293 cells transiently transfected with 1 ug of WT FLAG-tagged TUBA1A or with increased scalar concentration of mutated FLAG-tagged TUBA1A. GAPDH was used as loading control [reproduced with permission from “GeneDx”(1/31/23)].

**FIGURE 4:**
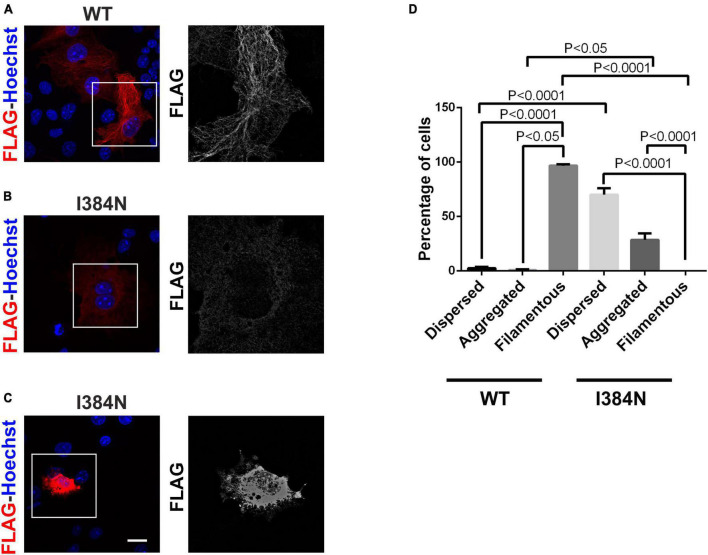
The p.I384N substitution prevents the incorporation of TUBA1A into microtubules. **(A)** Immunocytochemical analysis of COS-1 cells over-expressing WT FLAG-tagged TUBA1A showed that the WT tubulin forms filamentous structures, which are typical of microtubules. Scale bar: 25 μm. A high magnification of the selected area is shown on the right. Immunocytochemical analysis of COS-1 cells over-expressing mutated FLAG-tagged TUBA1A, showed that the TUBA1A harboring the p.I384N substitution is not incorporated into microtubules, appearing mainly dispersed in the cytoplasm **(B)** or in aggregated form **(C)**. Scale bar: 25 μm. A high magnification of the selected area is shown on the right. **(D)** The histogram shows the effect of the p.I384N substitution on TUBA1A state (dispersed, aggregated and filamentous). The percentage of each TUBA1A state was calculated as: [Number of each (TUBA1A state/Number of transfected cells) × 100]. The graph reports the mean ± SEM (250 cells from WT and 201 cells from p.I384N). Data were analyzed by one- way Anova [reproduced with permission from “GeneDx”(1/31/23]).

To analyze the functional impact of p.I384N substitution, WT and mutated N-terminally FLAG -tagged TUBA1A constructs were generated and transfected into HEK-293 cells. Immunoblot analysis were performed to assess the level of recombinant TUBA1A proteins. Quantitative western blotting revealed an approximate 94% decrease of the mutant TUBA1A as compared to the WT protein (Figure 3A), despite no differences in *TUBA1A* mRNA levels were observed between HEK-293 cells over-expressing WT and mutated tubulin (Figure 3B). To demonstrate that the reduction of TUBA1A mutant protein was not due to a lower transfection efficiency of the mutated construct, HEK-293 cells were co-transfected with expression vectors encoding for WT and mutated TUBA1A and with the same amount of green fluorescent protein (GFP) reporter construct, which was specifically used to determine the transfection efficiency of the experiment. As shown in Supplementary Figure 1A, while the protein levels of GFP were the same between HEK-293 cells over-expressing WT and mutant TUBA1A, the protein level of the mutated tubulin were specifically reduced. A drastic reduction of mutant TUBA1A protein level was also observed in transiently transfected COS-1 cells (Figure 3C) and E2GFP-D7 cells motor neuron progenitors (Figure 3D). To further prove the effect of the p.I384N substitution on TUBA1A stability, we also assessed the protein level of WT and mutated TUBA1A recombinant proteins in SH-SY5Y and HeLa cells. As shown in Supplementary Figures 1B, C immunoblot analysis confirmed the protein reduction of TUBA1A mutant also in these two addition cell lines. HEK-293 cells were also transfected using increasing scalar concentration of mutant TUBA1A construct. As shown in Figure 3E, even transfecting HEK-293 cells with higher concentration of mutant constructs, the protein levels of the mutated TUBA1A remained lower than the WT, thus suggesting that p.I384N mutation may affect the short-term protein stability of TUBA1A. To explore the effect of p.I384N substitution on microtubule architecture, COS-1 cells were transiently transfected to over-express WT and mutant TUBA1A constructs, and analyzed by immunocytochemical experiments. As shown in Figure 4, while WT TUBA1A formed filamentous structure, which are typical of microtubules (Figure 4A), mutated TUBA1A was depleted from microtubules, mainly appearing as dispersed, at low levels among the cytoplasm (Figure 4B). Interestingly, we also observed that in a low but significant percentage of cells, the p.I384N substitution increased TUBA1A propensity to form aggregates (Figures 4C, D). We indeed quantified the percentage of the TUBA1A state (classified as filamentous, dispersed and aggregated), observing that both dispersed and aggregated state of TUBA1A were almost exclusively found only in COS1-cells over-expressing TUBA1A mutant (Figure 4D). We also quantified the mean fluorescent intensity of mutated TUBA1A state (dispersed and aggregated), observing that it was significantly increased for TUBA1A aggregates, as compared to that of dispersed intensity (Supplementary Figure 2A).

We suppose that TUBA1A aggregation specifically occurs in cells whose proteolytic systems are overloaded, thus causing a buildup of mutated TUBA1A, which leads to an increase of its fluorescence intensity. However, although the high fluorescence levels of TUBA1A aggregates, the average of the mean fluorescence intensity of total TUBA1A state was still mildly lower in cells expressing the TUBA1A mutant (Supplementary Figure 2B).

Further, we evaluated the percentage of COS-1 cells over-expressing WT and mutated TUBA1A which were positive for the FLAG-staining, and accordingly to the reduced short-term stability of TUBA1A mutant, we observed that the percentage of FLAG-positive cells transfected with the mutated construct was significant lower as compared to that over-expressing the WT (39% for WT vs. 19% for p.I384N) (Supplementary Figure 2C). Our hypothesis is that TUBA1A mutant is efficiently degraded in a consistent percentage of cells, which accordingly appear negative for the FLAG staining, after immunofluorescence experiments. These findings explain the apparent discrepancy observed between the mean fluorescence intensity of TUBA1A mutant, which was mildly reduced as compared to that of WT (Supplementary Figure 2B), and its protein levels (Figure 3 and Supplementary Figure 1), which instead were drastically lower. Indeed, although the total mean fluorescence intensity of TUBA1A mutant was slightly reduced as compared to that of WT, COS-1 cells over-expressing the mutated tubulin were still lower as compared to that over-expressing the WT tubulin, hence the immunoblot analysis, which is representative of TUBA1A protein levels of total transfected cells, showed a relevant reduction of TUBA1A mutant.

Consistent with the reduced stability and the aggregation propensity of TUBA1A^*I*384*N*^, nocodazole wash out experiments showed that TUBA1A mutant fails to assembly into microtubule network *in vitro* (Supplementary Figures 3A, B). Accordingly, experiments of microtubule partitioning into soluble and polymerized fractionation showed that mutated TUBA1A localizes exclusively in the soluble fraction, corresponding to unpolymerized tubulins (Supplementary Figure 3C).

Further, we examined the overall amount and the structure of endogenous microtubules. As shown in Supplementary Figures 4, 5, both immunoblot and immunocytochemical analysis of transfected cells, revealed that the over-expression of TUBA1A mutant does not perturb either the protein levels of endogenous α and β-tubulins or the organization of endogenous microtubules. However, these findings cannot exclude that in physiological condition, the reduce availability of TUBA1A may affect the overall amount of tubulins and, accordingly, the structure of microtubule cytoskeleton, and that such reduction does not occur in transfected cells, since the endogenous α-tubulin compensates for the lack of transfected TUBA1A.

**FIGURE 5:**
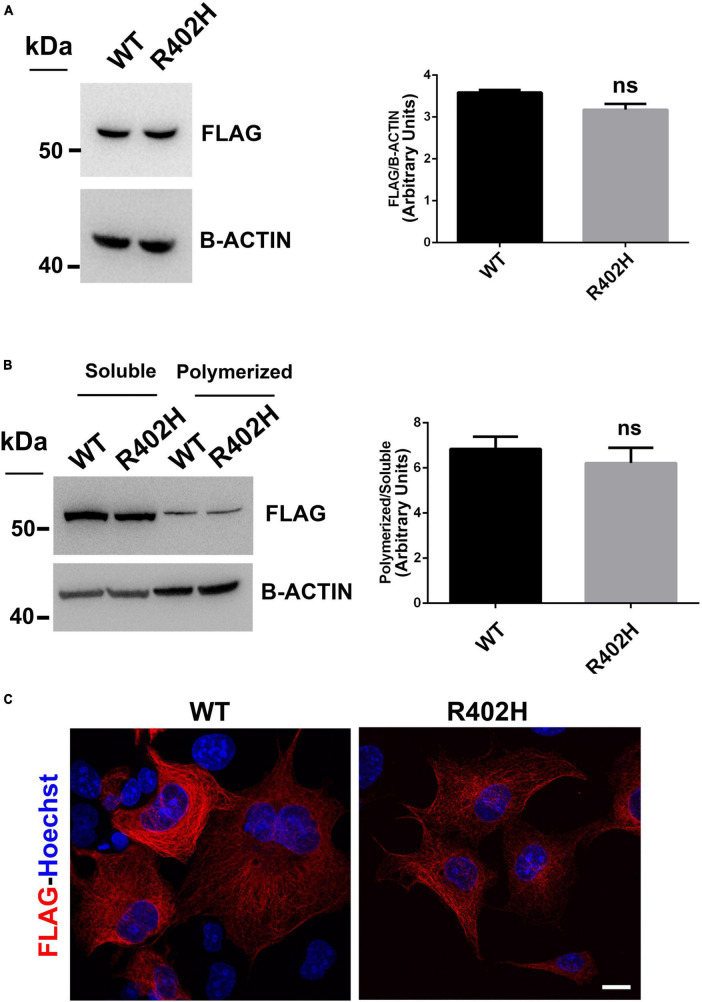
The R402H substitution, previously reported to cause lissencephaly, does not perturb TUBA1A stability or incorporation into microtubules. **(A)** Immunoblot analysis of HEK-293 cells transiently transfected with FLAG-tagged TUBA1A (WT and mutant) did not documented changes in the protein level of TUBA1A^WT^ and TUBA1A^R402H^. β-actin was used as loading control. Histogram shows the quantitative analysis for western blot. The graph reports the mean ± SEM. Data were analyzed by Mann Whitney test *N* = 3. **(B)** Immunoblot analysis of soluble and polymerized pools of TUBA1A in HEK-293 cells overexpressing TUBA1A^WT^ and TUBA1A^R402H^. β-actin was used as loading control to demonstrate that soluble fractions were equally loading among them and that polymerized fractions were equally loading among them. Histogram shows the quantitative analysis for western blot. The graph reports the mean ± SEM. Data were analyzed by Mann Whitney test *N* = 3. **(C)** Immunocytochemical analysis of COS-1 cells over-expressing FLAG-tagged TUBA1A (WT and mutant) showed that mutated tubulin was correctly incorporated into microtubules. Scale bar: 25 μm.

We also examined the impact of the p.R402H substitution, which has been previously associated to severe lissencephaly (Yokoi et al., 2015), on TUBA1A stability and microtubule incorporation. As shown in Figure 5, we found that the p.R402H mutation does not perturb TUBA1A protein stability, since no significant reduction of mutated TUBA1A was observed, compared to the WT (Figure 5A).

Moreover, immunoblot analysis of soluble and polymerized pools of TUBA1A isolated from HEK-293 cells over-expressing TUBA1A*^WT^* or TUBA1A^*R*402*H*^, showed a comparable polymerization rate of WT and mutant protein (Figure 5B), indicating that TUBA1A^*R*402*H*^ is assembled into microtubules. Accordingly, immunocytochemical analysis of transfected COS-1 cells confirmed that TUBA1A mutant was correctly incorporated into microtubule lattice (Figure 5C).

### 3.5. The pathogenic effect of p.I384N substitution is independent of the tubulin isoforms

Since the isoleucine in position 384 is highly conserved among α-tubulins (Supplementary Figure 6), we decided to verify if the pathogenic effect of the p. I384N substitution was specific for TUBA1A or could occur in other α-tubulins. To answer this question FLAG-tagged TUBA1B*^WT^*, FLAG-tagged TUBA4A*^WT^* and the corresponding mutated constructs were generated and over-expressed into HEK-293 and COS-1 cells.

**FIGURE 6:**
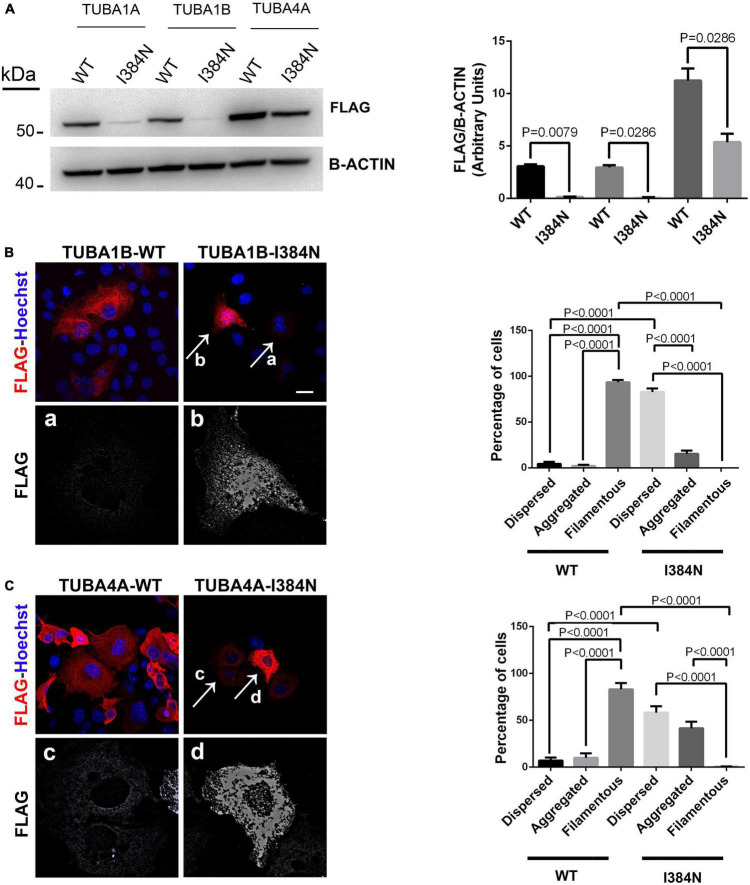
The Isoleucine in position 384 is an amino acid residue critical for tubulin stability.**(A)** Immunoblot analysis of HEK-293 cells over-expressing WT or mutated FLAG-tagged TUBA1A, WT or mutated FLAG-tagged TUBA1B and WT or mutated FLAG-tagged TUBA4A recombinant proteins harboring the p.I384N substitution, revealed a significant reduction of all mutated tubulins. β-actin was used as loading control. Histogram shows the quantitative analysis for western blot. The graph reports the mean ± SEM. Data were analyzed by Mann Whitney test *N* = 4. **(B)** Immunocytochemical analysis of COS-1 cells over-expressing WT or mutated FLAG-tagged TUBA1B recombinant protein showed that the p.I384N substitution prevents the incorporation of the mutated tubulin into microtubules and increase its aggregation propensity. Scale bar: 25 μm. A high magnification of cells indicated by the arrows shows the mutated TUBA1B in its dispersed (panel a) and aggregated (panel b) state. The histogram on the right shows the effect of the p.I384N substitution on TUBA1B state (dispersed, aggregated and filamentous). The percentage of each TUBA1B state was calculated as: [Number of each (TUBA1B state/Number of transfected cells) × 100]. The graph reports the mean ± SEM (206 cells from WT and 202 cells from p.I384N). Data were analyzed by one way Anova. **(C)** Immunocytochemical analysis of COS-1 cells over-expressing WT or mutated FLAG-tagged TUBA4A recombinant protein showed that the p.I384N substitution prevents the incorporation of the mutated tubulin into microtubules and increase its aggregation propensity. Scale bar: 25 μm. A high magnification of cells indicated by the arrows shows the mutated TUBA4A in its dispersed (panel c) and aggregated (panel d) state. The histogram on the right shows the effect of the p.I384N substitution on TUBA4A state (dispersed, aggregated, and filamentous). The percentage of each TUBA4A state was calculated as: [Number of each (TUBA4A state/Number of transfected cells) × 100]. The graph reports the mean ± SEM (207 cells from WT and 208 cells from p.I384N). Data were analyzed by one way Anova [reproduced with permission from “GeneDx”(1/31/23)].

As shown in Figure 6A, immunoblot analysis revealed that all tubulin isotypes harboring the p.I384N substitution were depleted from cell lysates of transfected HEK-293 cells. Moreover, immunofluorescence experiments conducted on transfected COS-1 cells, showed that both mutated TUBA1B and TUBA4A proteins were not incorporated into microtubules appearing as puncta that were diffusely distributed throughout the cytoplasm (Figure 6B-panel a, Figure 6C-panel c). Moreover, mutated TUBA1B and TUBA4A isoforms also showed an increased propensity to aggregate in the cytosol (Figure 6B-panel b, Figure 6C-panel d), which was particularly evident for the TUBA4A mutated protein (Figure 6C).

These data demonstrate that the pathogenic effect of the p.I384N mutation is independent of the tubulin isoforms and not restricted to the *TUBA1A* gene.

### 3.6. The p.I384N and N102 substitutions affect TUBA1A proteostasis and trigger TUBA1A proteasome-dependent degradation

Recently, the loss of function p.N102D substitution, in heterozygous state in Tuba1a has been reported to reduce Tuba1a and developmental total α-tubulin stability, causing adult onset movement disorder and ataxia in *Tuba1a^ND/+^*mice (Buscaglia et al., 2020, 2022).

We decided to investigate if both p.N012D and p. I384 mutations may cause neurodegeneration through common pathogenic mechanisms.

Since mouse and human TUBA1A proteins present the same amino acid change, we introduced the p.N102D substitution into human WT FLAG-tagged TUBA1A and then used it to perform a comparative analysis between p.I384N and N102D mutations.

**FIGURE 7:**
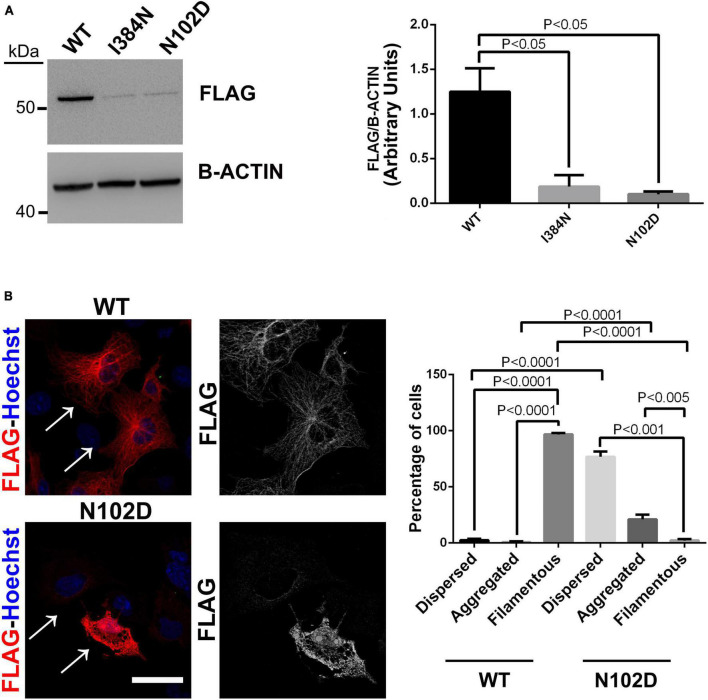
The heterozygous p. N102D substitution, previously reported to cause adult-onset ataxia in *Tuba1a^ND/+^*mice, affects TUBA1A protein stability and prevents its incorporation into microtubules. **(A)** Immunoblot analysis of HEK-293 cells transiently transfected with WT or mutated FLAG- tagged TUBA1A recombinant proteins harboring the p.N102D and the p.I384N substitutions, documented a similar reduction of both mutated tubulin. β-actin was used as loading control. Histogram shows the quantitative analysis for western blot. The graph reports the mean ± SEM. Data were analyzed by Kruskal-Wallis test and analyzed *post-hoc* using the Dunn’s test *N* = 5. **(B)** Immunocytochemical analysis of COS-1 cells over-expressing FLAG-tagged TUBA1A (WT and N102D mutant) showed that the p.N102D substitution prevents TUBA1A incorporation into microtubules and increases its aggregation propensity. Scale bar: 25 μm. A high magnification of cells indicated by the arrows is shown on the right The histogram on the right show the effect of the p.I384N substitution on TUBA1A state (dispersed, aggregated, and filamentous). The percentages of each TUBA1A state were calculated as: [Number of each (TUBA4A state/Number of transfected cells) × 100]. The graph reports the mean ± SEM (250 cells from WT and 200 cells from p.N102D). Data were analyzed by one-way Anova [reproduced with permission from “GeneDx”(1/31/23)].

As previously reported by Buscaglia et al. (2022), and similarly to results we obtained for the mutant TUBA1A^*I*384*N*^, immunoblot and immunocytochemical analysis showed that TUBA1A^*N*012*D*^ was depleted from the cell lysate and from microtubules of transfected HEK-293 and COS-1 cells, respectively (Figures 7A, B). Moreover, as observed for the p.I384N mutation, we also found that the p.N102D substitution increased TUBA1A aggregation in a low percentage of cells (Figure 7B).

**FIGURE 8:**
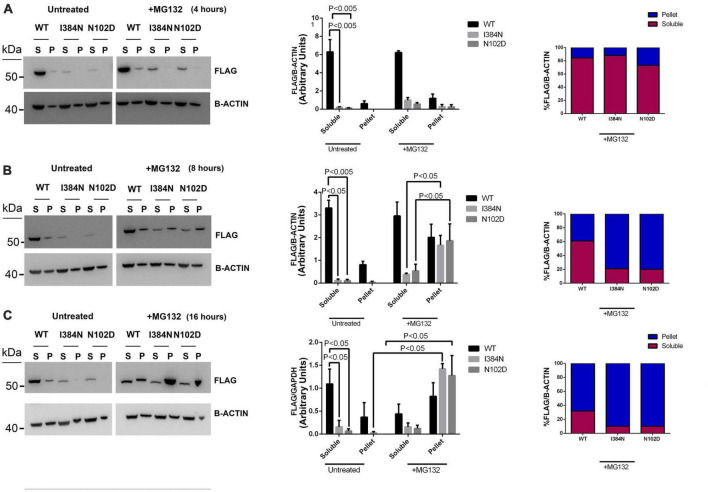
Proteasome inhibition causes the redistribution of TUBA1A mutants from soluble to insoluble fraction, in a faster mode compared to WT. Immunoblot analysis of HEK cells over-expressing TUBA1A^WT^, TUBA1A ^I384N^, TUBA1A^N102D^ and treated for 4 **(A)**, 8 **(B)**, and 16 **(C)** hours with the proteasome inhibitor MG132 or with DMSO (untreated), revealed that the block of the proteasome system increases the accumulation of mutated TUBA1A proteins from the detergent-soluble (S) to the detergent-insoluble (P) fraction, in a faster mode as compared to TUBA1A^WT^. **(A–C)** Histograms on the left show the quantitative analysis for each western blot. β-actin was used as loading control. The graph reports the mean ± SEM. Data were analyzed by Kruskal-Wallis test and analyzed *post-hoc* using thr Dunn’s test **(A,C)** *N* = 3 **(B)** *N* = 4. Histograms on the right show the percentage of TUBA1A (WT and mutated) in each protein fraction (Soluble and Pellet) [reproduced with permission from “GeneDx”(1/31/23)].

**FIGURE 9:**
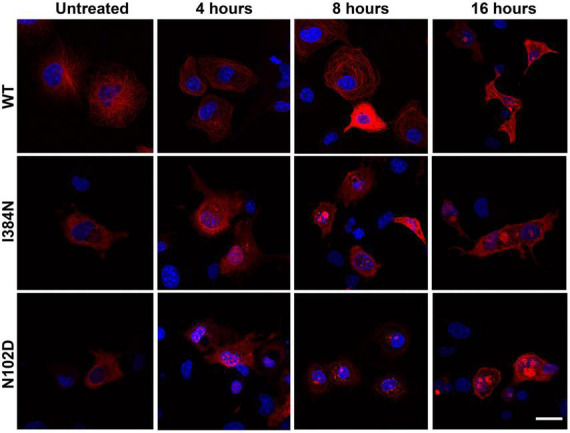
Proteasome inhibition favors the accumulation of TUBA1A^I384N^ and TUBA1A^N102D^ into aggregated conformers and cytosolic inclusions. Immunocytochemical analysis of COS-1 cells over-expressing TUBA1A^WT^, TUBA1A ^I384N^, TUBA1A^N102D^ and treated for 4, 8, and 16 h with MG132, showed that the block of the proteasome increases the accumulation of TUBA1A mutants in their aggregated forms and their accumulation into inclusions. Prolonged time of proteasome inhibition (16 h) induces the accumulation of TUBA1AWT into aggregated conformers. Scale bar: 25 μm [reproduced with permission from “GeneDx”(1/31/23)].

We also evaluated the contribution of the proteasome degradative system, which is the major intracellular proteolytic pathway, on the degradation of TUBA1A^*I*384*N*^ and TUBA1A^*N*012*D*^ mutated proteins. For this reason, HEK-293 and COS-1 cells over-expressing WT or mutated recombinant tubulins were treated, for different times, with the proteasome inhibitor MG132 and analyzed by western blotting and immunofluorescence, respectively. As shown in Figure 8A, after 4 h of MG132 administration, both TUBA1A mutants were mainly detected in the soluble-detergent fraction of HEK-293 cells and significantly increased in this portion (3.6-fold of increase for TUBA1A^*I*384*N*^ and TUBA1A^*N*102*D*^) (Figure 8A). Moreover, immunocytochemical analysis of transiently transfected COS-1 cells treated for 4 h with MG132, revealed the accumulation of both mutant tubulins in aggregated form (Figure 9). Similarly, at the same time of MG132 administration, TUBA1A*^WT^* was mainly detected in the soluble fraction and protein levels of this portion were almost unchanged as compared to the untreated. A moderate but not significant increase of TUBA1A insoluble form was also observed (1.9-fold of increase) (Figure 8A). Despite this finding, immunofluorescence analysis of COS-1 cells over-expressing TUBA1A*^WT^* did not detect the presence of tubulin aggregates, after 4 h of MG132 treatment (Figure 9). Instead, after 8 h of MG132 administration, both TUBA1A mutants were almost totally detected in their insoluble forms (80% insoluble vs. 20% soluble for both TUBA1A^*I*384*N*^ and TUBA1A^*N*102*D*^) (Figure 8B). Furthermore, immunocytochemical analysis of COS-1 cells over-expressing both mutated proteins and treated for 8 h with MG132, revealed the formation of TUBA1A inclusions (Figure 9). Although after 8 h of proteasome inhibition, a consistent portion of TUBA1A*^WT^* was found to localize in the detergent insoluble fraction (39% insoluble vs. 61% soluble), the percentage of insoluble TUBA1A*^WT^* was anyway lower as compared to that of mutated TUBA1A proteins (80% insoluble vs. 20% soluble for both TUBA1A^*I*384*N*^ and TUBA1A^*N*102*D*^) (Figure 8B). Moreover, immunofluorescence analysis of transfected COS-1 cells showed that, after 8 h of MG132 treatment, TUBA1A*^WT^* mainly appears in its polymerized state, even though the formation of tubulin aggregates was also observed (Figure 9). After prolonged times of proteasome inhibition (16 h), immunoblot analysis of transfected HEK-293 cells, revealed that TUBA1A*^WT^* was mainly redistributed in the cell pellet (68% insoluble vs. 32% soluble) (Figure 8C). However, immunocytochemical analysis of COS-1 cells showed that TUBA1A*^WT^* retains, although to a small extent, its ability to polymerize. However, the microtubule network in these cells appeared to be disorganized and the formation of tubulin aggregates and inclusions were also observed (Figure 9). Differently, after 16 h of MG132 administration, TUBA1A mutant proteins, were almost totally found to localize in the detergent insoluble fraction (90% insoluble vs. 10% soluble for both TUBA1A^*I*384*N*^ andTUBA1A^*N*102*D*^) (Figure 8C) and was totally detected in the form of inclusions (Figure 9). We also evaluated by qRT-PCR, *TUBA1A* expression levels of transiently transfected HEK-293 cells, at different times of MG132 treatment, without observing any changes in mRNA levels of WT and mutated *TUBA1A* (Supplementary Figure 7). These evidences further confirm that the faster accumulation of TUBA1A mutant, after MG132 treatment is indeed caused by the lacking of its proteasomal degradation and not by variations in its gene expression. Moreover, we evaluated the percentage of COS-1 cells over-expressing TUBA1A*^WT^* and TUBA1A^*I*384*N*^ which were positive for the FLAG-staining, after MG132 treatment. We observed that while the percentage of cells transfected with the WT construct was almost the same before and after 4 and 8 h of MG132 treatment, the percentage of cells transfected with the mutated construct were increased, as compared to the untreated (Supplementary Figure 8). After 16 h of MG132 treatment, a slight decrease in the percentage of both WT and mutated cells, as compared to the previously time of treatments, was observed. This reduction could be caused by the prolonged times of MG132 treatment, which may be toxic for cells. However, the transfection levels of TUBA1A mutant remain anyway, increased, as compared to those of untreated cells (Supplementary Figure 8).

## 4. Discussion

Mutations in tubulin genes are associated with a wide spectrum of neurological disorders collectively known as “tubulinopathies” and mostly characterized by cortical and subcortical malformations (Breuss et al., 2012; Poirier et al., 2013; Bahi-Buisson et al., 2014; Cushion et al., 2014; Brock et al., 2018; Romaniello et al., 2018; Sferra et al., 2020).

Despite tubulin mutations have been mainly linked to neurodevelopmental defects, there is growing evidence that perturbations of tubulin functions and activities can also drive neurodegeneration (Tischfield et al., 2013; Smith et al., 2014; Sferra et al., 2016, 2018).

Heterozygous mutations in *TUBA1A* gene, which encodes for one of the major neuronal α-tubulins, has been associated, so far, with severe brain malformations including lissencephaly, cerebellar hypoplasia, agenesis of the corpus callosum, and brain stem anomalies (Keays et al., 2007; Poirier et al., 2007; Kumar et al., 2010; Yokoi et al., 2015; Hebebrand et al., 2019).

In the present study, we report on the novel loss-of-function mutation p.I384N in TUBA1A, resulting in progressive spastic paraplegia and ataxia.

We investigated the pathogenic effect of the disease causing-mutation, demonstrating that it occurs by affecting TUBA1A proteostasis and reducing the abundance of TUBA1A protein, available in the cell (Figures 3, 4).

In contrast to the p.R402H mutation, which has been reported to cause lissencephaly (Yokoi et al., 2015) without impairing either TUBA1A protein homeostasis or assembly into microtubules (Figure 5), we showed the p.I384N substitution impairs TUBA1A stability, preventing its incorporation into the lattice (Figures 3, 4). In HEK-293, COS-1 and E2GFP-D7 cells motor neuron progenitor over-expressing TUBA1A^*I*384*N*^ a drastic reduction of mutant TUBA1A protein level was observed (Figure 3). Moreover, in transfected COS-1 cells, the mutant tubulin was not incorporated into microtubules, and was mainly detected at low levels as puncta that were diffusely distributed throughout the cytoplasm (Figure 4B). Interestingly, we also found that, although in a low percentage of cells, TUBA1A^*I*384*N*^ forms cytosolic aggregates as well (Figures 4C, D).

Thus, although the present disease-causing mutation is a missense variant not predicting RNA degradation, it acts with a haploinsufficiency mechanism since it reduces the availability of TUBA1A within the cells, and its pathogenic effect is comparable to that of a loss of function, rather than a gain of function mutation.

The isoleucine in position 384 is a highly conserved residue involved in both polar and hydrophobic interactions which are critical for TUBA1A structure, and the p.I384N variant was predicted to destabilize the TUBA1A protein, since the replacing asparagine differs in both polarity and size (Figure 2). Since the mutated isoleucine is highly conserved among α-tubulins (Supplementary Figure 6) we enquired whether the effect of I384N substitution was specific for TUBA1A or generally affected α-tubulins. Immunoblot and immunocytochemical analysis of TUBA1A, TUBA1B, and TUBA4A proteins harboring the p.I384N mutation revealed that all mutant tubulins were significantly depleted from total protein extracts and from microtubules of transfected cells, as compared to their respective WT, thus demonstrating that the isoleucine at 384 position is critical for the stability of also additional α-tubulins as well (Figure 6).

Recently, the heterozygous p.N102D substitution in *Tuba1a* gene identified by ENU-induced mutagenesis forward genetic screening in mice with locomotor defects, has been reported to cause adult-onset movement disorder and ataxia (Buscaglia et al., 2020, 2022). Interestingly, this pathogenic variant has been recently demonstrated to decrease α-tubulin protein levels in yeast and *Tuba1a^ND/+^*mice, reducing the incorporation of the mutant α-tubulin into microtubule lattice (Gartz Hanson et al., 2016; Buscaglia et al., 2020, 2022) and causing the formation of less stable microtubules that are insufficient to support axonal trafficking, during the early development of *Tuba1a^ND/+^* mice (Buscaglia et al., 2022).

Our comparative analysis of p.N102D and p.I384N substitutions showed that both mutant proteins were excluded from protein extract and from microtubules of transfected cells (Figure 7). Moreover, similarly to p.I384N, we also found that the p.N102D mutation increased TUBA1A propensity to form protein aggregates (Figure 7B). Since both p.I384N and p.N102D substitutions reduce the availability of TUBA1A within the cell preventing its assembly into microtubule lattice, we hypothesize, as previously reported for the heterozygous p.N102D mutation (Buscaglia et al., 2020, 2022), that in neurons harboring the p.I384N substitution, TUBA1A protein level may be not enough to allow the proper tubulin polymerization and that microtubules may be not correctly set up, thus limiting cargo run length and increasing pause times of motor proteins.

Structural features of microtubules, such as their length and thickness within the network, indeed, affect the kinetic of axonal transport. Motor proteins pause at microtubule ends before switching to a new polymer, and the pause time that they spent in the immobile state depend on microtubule density. In addition, cargo run length within the axon overlap with microtubule length (Yogev et al., 2016).

Further, *in vivo* studies have demonstrated that tubulin isoforms are not functionally interchangeable (Saillour et al., 2014) and according to this evidence, gene expression analysis revealed that in developing *Tuba1a^ND/+^* mouse brain, alternative α-tubulins are not upregulated to compensate for the loss of TUBA1A protein (Buscaglia et al., 2020). Hence, it is captivating to hypothesize that in neurons harboring the heterozygous p.I384N substitution, *TUBA1A WT* allele may fail to compensate for the reduced α-tubulin, and, as a consequence, *TUBA1A* deficient neurons may be more prone to degenerate because microtubules cannot support any effective axonal transport.

We also demonstrated that mutant TUBA1A^*I*384*N*^ and TUBA1A^*N*102*D*^ proteins are degraded by the proteasome, since the block of this proteolytic pathway increased their protein levels, favoring the formation of cytosolic aggregates of TUBA1A mutants that accumulate into the insoluble protein fraction faster as compared to TUBA1A*^WT^* (Figures 8, 9). Therefore, another not-exclusive possibility is that the deleterious effect of p.I384N and p.N102D mutations may occur by overburdened cellular proteolytic systems belatedly in post mitotic neurons, which are more sensitive to the accumulation of cytotoxic inclusions.

Interestingly, a similar pathogenic mechanism has been proposed for the p.W407X substitution in TUBA4A, which has been associated to a severe phenotype of amyotrophic lateral sclerosis (Smith et al., 2014). Moreover, the redistribution of microtubule elements from the soluble to insoluble fraction or their organization into aggregated conformers have been yet observed in different *in vitro* models of Parkinson’s disease (Alim et al., 2002; Diaz-Corrales et al., 2005; Cartelli et al., 2016; Seebauer et al., 2022).

In H4 neuroglioma cells over-expressing α-synuclein and in midbrain dopaminergic neuronal cells obtained from patients harboring α-synuclein duplication, the increase of α-synuclein was accompanied by a shift of soluble class III β-tubulin into the insoluble protein fraction (Seebauer et al., 2022), and the intranuclear inclusions observed in neurons of human brain of patients affected by Parkinson’s disease have been reported to be immunoreactive to class III β-tubulin (Alim et al., 2002). Moreover, Cartelli et al. (2016) have demonstrated that different α-synuclein variants related to Parkinson’s disease do not undergo tubulin-induced folding and cause tubulin aggregation. Gamma tubulin aggregation was also found in Lewy bodies of Parkinson’s disease and recently in rotenone treated neurons, a validated experimental model of this disease (Diaz-Corrales et al., 2005).

However, since *in silico* and functional analysis demonstrated that the p.I384N substitution drastically affects TUBA1A stability, it can’t be excluded that the present disease causing mutation may also limit the chaperone-dependent formation of tubulin heterodimers by perturbing the generation of folding intermediate. Experiments of tubulin partitioning, has shown that TUBA1A mutant was exclusively found in its unpolymerized state (Supplementary Figure 3C) and short-time inhibitions of the proteasome, which induces a moderate increase of TUBA1A mutant in the detergent-soluble fraction (Figure 8A), does not correlate with the formation of filamentous structures (Figure 9), thus suggesting that the small amount of mutated TUBA1A available in the cell, effectively fails to polymerize. However, our data are currently insufficient to prove if the mutated TUBA1A may limit the pool of functional heterodimers and further experiments will be necessary to verify it and eventually identify which steps of the overall pathway of heterodimer formation, are impaired.

Nevertheless, Wang et al. (2006) have demonstrated that mutations that destabilize the folding of β-1 tubulin, perturbing its stability and causing its rapid proteasomal degradation, as we observed for the p.I384N substitution, lead to a greatly reduced amount of functional heterodimers, by impairing the interaction between mutated tubulins and cofactors involved in the heterodimer folding pathway.

Lastly, Miyasaka et al. (2018) have recently demonstrated that the knockdown of subset of tubulins led to enhanced tau toxicity in pan-neurons of *C-elegans*, and that tau aggregation was inhibited by co-incubation of purified tubulin *in vitro*, indicating that the proper amount of tubulins is critical to prevent tau self-aggregation. Therefore it cannot be excluded that the decline of TUBA1A may also trigger tau toxicity in neurons. In conclusion, in the present study we associate a novel loss of function mutation in *TUBA1A* to a neurodegenerative phenotype characterized by progressive spastic paraplegia and ataxia, by expanding both phenotypic and mutational spectrum of *TUBA1A*. Moreover, our data suggest that the reduced availability of TUBA1A, due to its increased protein instability and proteostasis, may be enough to induce neurodegeneration in humans.

## Data availability statement

The datasets presented in this study can be found in online repositories. The names of the repository/repositories and accession number(s) can be found in the article/Supplementary material.

## Ethics statement

The studies involving human participants were reviewed and approved by the Ospedale Pediatrico Bambino Gesù (2356_OPBG_RC_2020, 04/28/2021), Bambino Gesù Children’s Hospital, IRCCS, Rome, Italy. Written informed consent to participate in this study was provided by the participants’ legal guardian/next of kin.

## Author contributions

AS designed the research, analyzed the experiments, and wrote the original draft. RZ performed the experiments and acquired the data. MP, VD’O, and SP performed the confocal analysis. EB performed the *in silico* analysis. KB performed the clinical anaysis. RS performed the experiments of motor neuron transfection. AS and EnB edited the manuscript and added comments. All authors read and agreed with the published version of the manuscript.

## Footnotes

1
www.pymol.org
