# Supplementary material for: Novel loss of function mutation in TUBA1A gene compromises tubulin stability and proteostasis causing spastic paraplegia and ataxia
Source: Front Cell Neurosci. 2023 Jun 23;17:1162363. doi: 10.3389/fncel.2023.1162363 (PMC10332271; doi:10.3389/fncel.2023.1162363)
Supplement: Supplementary file 1 [file Data_Sheet_1.docx]

Supplementary Material

Novel loss of function mutation in *TUBA1A* gene compromises tubulin stability and proteostasis causing spastic paraplegia and ataxia

Riccardo Zocchi^1^, Emanuele Bellacchio^2^, Michela Piccione^3^, Raffaella Scardigli^4,5^, Valentina D’Oria^3^, Stefania Petrini^3^, Kristin Baranano^6^, Enrico Bertini^1^, Antonella Sferra^1^*

*** Correspondence:** Antonella Sferra: [antonella.sferra@opbg.net](mailto:antonella.sferra@opbg.net)

## Supplementary Figures


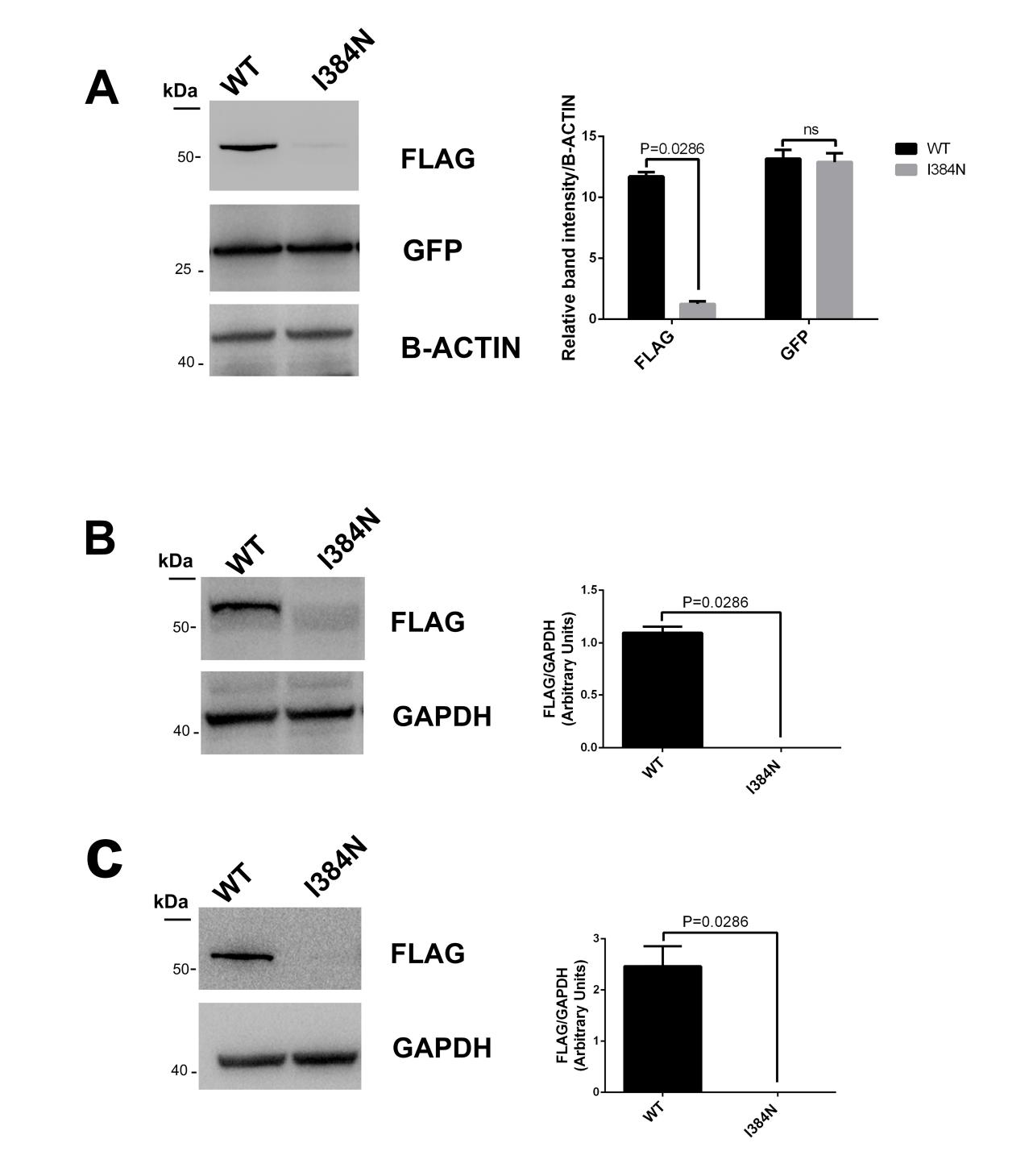


**Supplemetary Figure 1.** The p.I384N substitution impairs TUBA1A protein stability **(A)** Immunoblot analysis of HEK-293 cells co-transfected with the same amount (1ug) of TUBA1A (WT and mutant) and GFP constructs, showed that the protein levels of the mutated tubulin were specifically reduced. The protein levels of GFP, which was used to determine the transfection efficiency of the experiment, did not vary among HEK-293 cells over-expressing the WT and mutant TUBA1A. Histogram shows the quantitative analysis for western blot. The graph reports the mean ± SEM. Data were analyzed by Mann Whitney test. N=4. (B) Immunoblot analysis of SH-SY5Y cells transiently transfected with FLAG-tagged TUBA1A (WT and mutant) documented a significant reduction of mutant tubulin. GAPDH was used as loading control. Histogram shows the quantitative analysis for western blot.The graph reports the mean ± SEM. Data were analyzed by Mann Whitney test. N=4. **(B)** Immunoblot analysis of HeLa cells transiently transfected with FLAG-tagged TUBA1A (WT and mutant) documented a significant reduction of mutant tubulin. GAPDH was used as loading control. Histogram shows the quantitative analysis for western blot.The graph reports the mean ± SEM. Data were analyzed by Mann Whitney test. N=4.


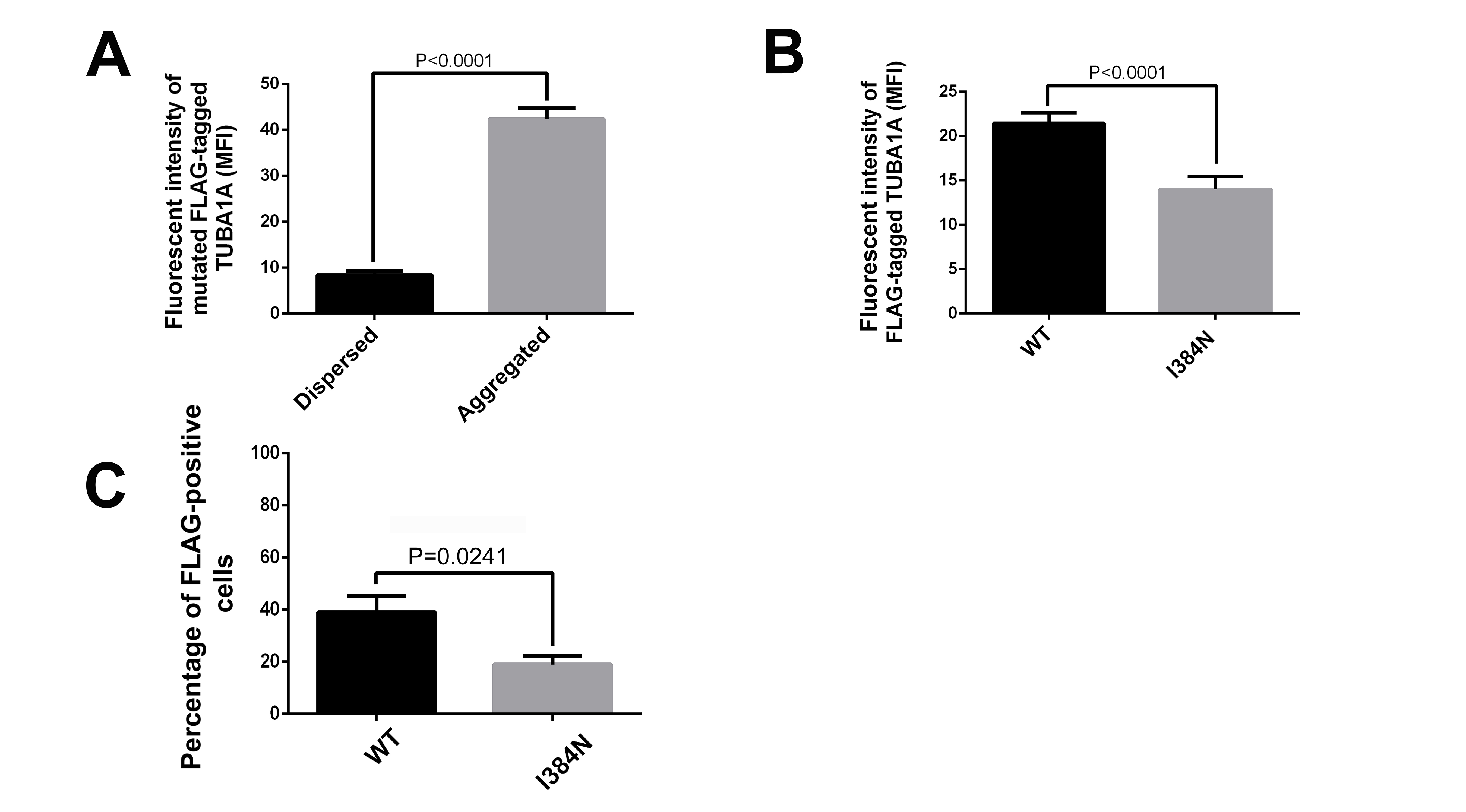


**Supplemetary Figure 2.** The p.I384N substitution affects the mean fluorescent intensity and the percentage of FLAG-positive cells over-expressing FLAG-tagged TUBA1A. **(A)** The histogram shows the mean fluorescent intensity (MFI) of mutated TUBA1A in its aggregated and dispersed state. The MFI was calculated using ImageJ/FIJI software (NIH) . The graph reports the mean ± SEM (38 cells for dispersed TUBA1A and 31 cells for aggregated TUBA1A). Data were analyzed by Mann Whitney test. **(B)** The histogram shows the average of MFI of WT and mutated TUBA1A state. The MFI was calculated using ImageJ/FIJI software (NIH) . The graph reports the mean ± SEM (77 cells from WT and 73 cells from p.I384N). Data were analyzed by Mann Whitney test. **(C)** The histogram shows the percentage of FLAG-positive COS-1 cells over-expressing WT and mutated TUBA1A, calculated as: (Number of FLAG positive cells/Number of total cell) x 100, of WT and mutated TUBA1A. The graph reports the mean ± SEM (587 cells from WT and 319 cells from p.I384N). Data were analyzed by Mann Whitney test (reproduced with permission from 'GeneDx '(1/31/23 )).


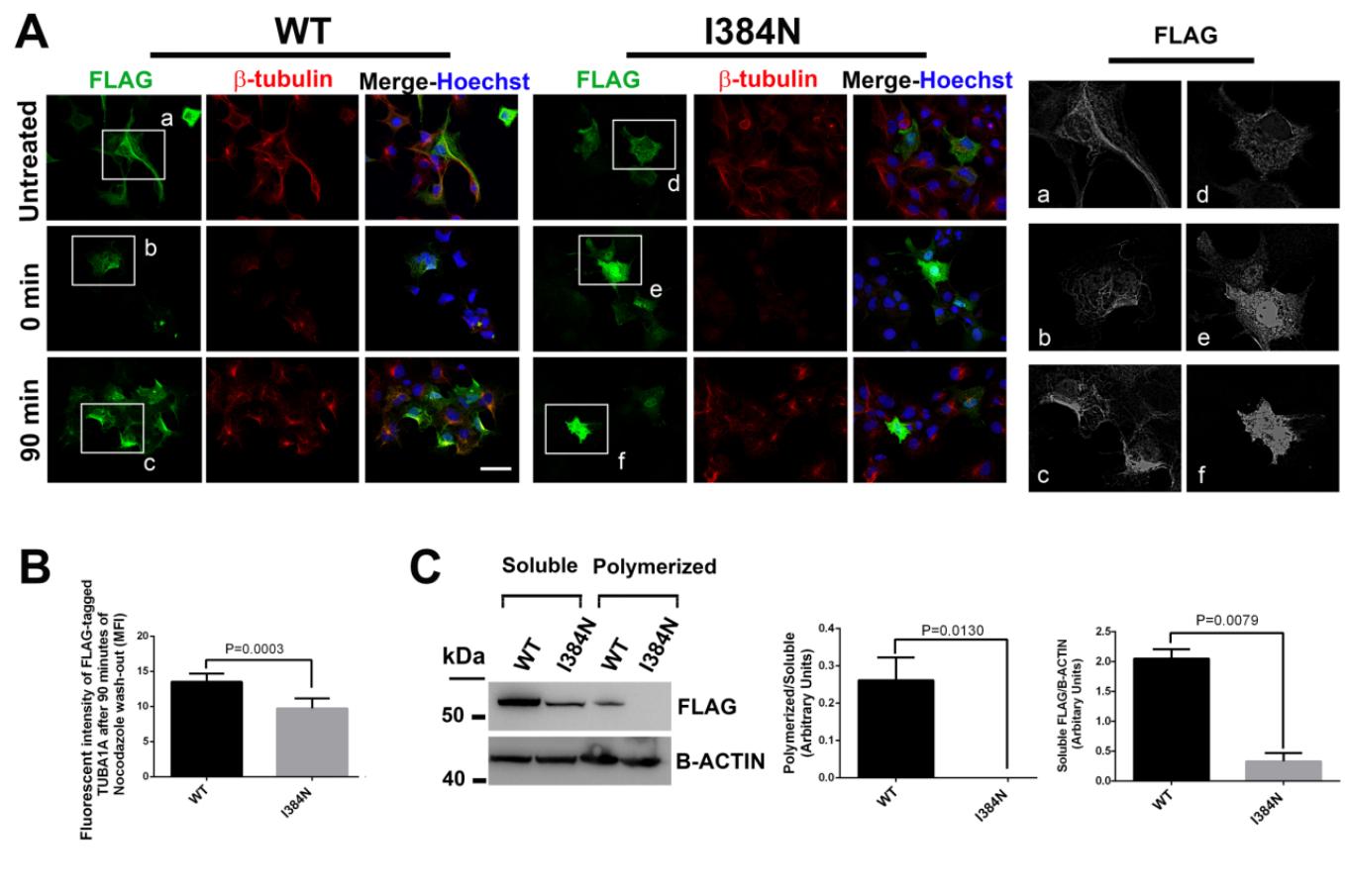


**Supplementary Figure 3.** TUBA1A harboring the p.I384N mutation is not incorporated into microtubules. **(A)** COS-1 cells over-expressing FLAG-tagged TUBA1A (WT and mutant) were treated with nocodazole (10 uM) for 45 minutes to induce microtubule depolymerization. After nocodazole wash-out, COS-1 cells were fixed with ice-cold methanol at the indicated times and stained for FLAG (green) and β-tubulin (red) to visualize the rate of TUBA1A and β-tubulin growth. Scale bar: 50 µm. On the right, a high magnification of the selected area. **(B)** The histogram shows the MFI of WT and mutated TUBA1A at 90 minutes, after nocodazole wash-out. The MFI was calculated using I ImageJ/FIJI software (NIH). The graph reports the mean ± SEM (73 cells from WT and 52 cells from p.I384N). Data were analyzed by Mann Whitney test. **(C)** Immunoblot analysis of soluble and polymerized pools of TUBA1A in HEK-293 cells over-expressing TUBA1A^WT^ and TUBA1A^I384^. β-actin was used as loading control to demonstrate that soluble fractions were equally loading among them and that polymerized fractions were equally loading among them. Histograms show the quantitative analysis for western blot. The graph reports the mean ± SEM. Data were analyzed by Mann Whitney test. N=5 (reproduced with permission from 'GeneDx '(1/31/23 )).

**
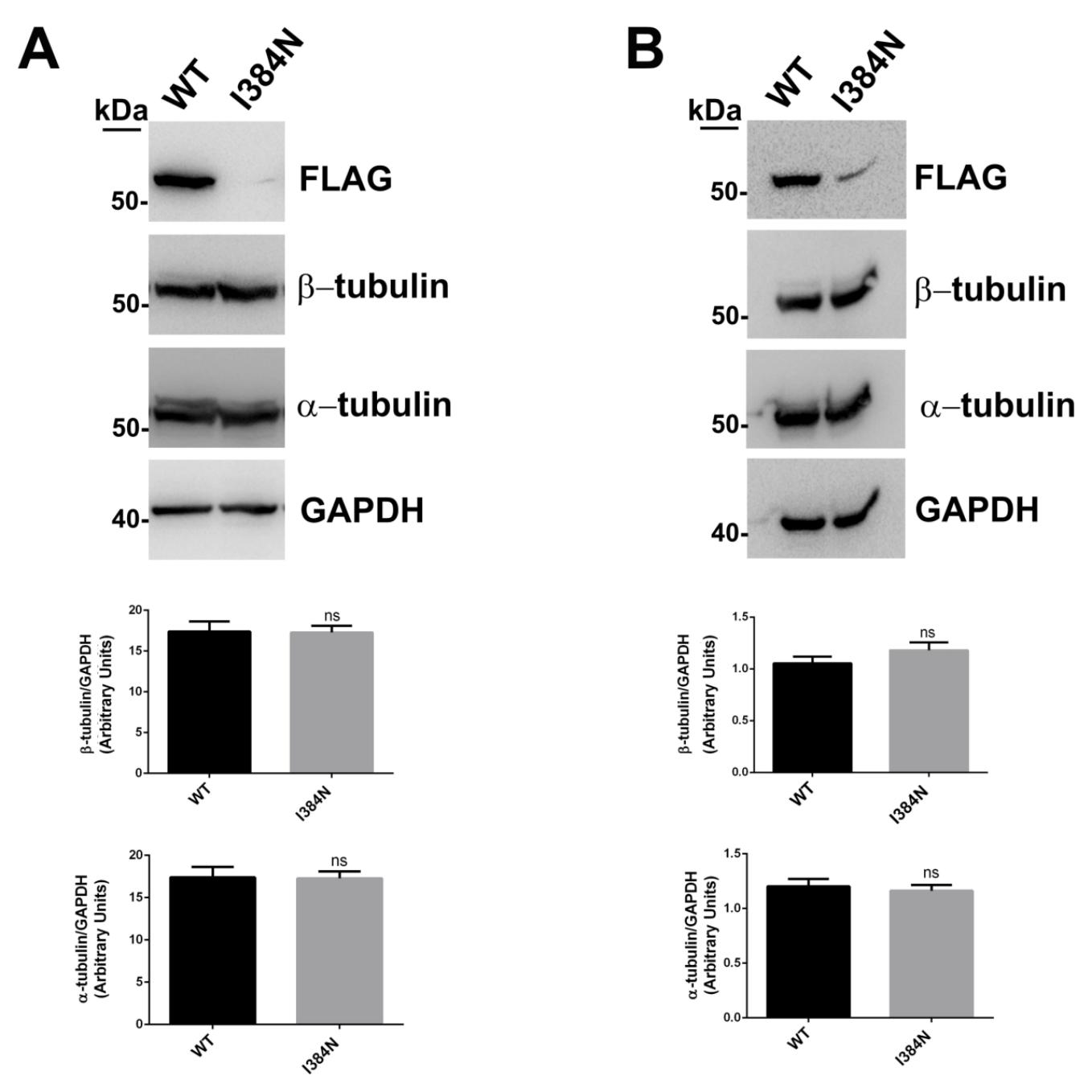
**

**Supplementary Figure 4.** The p.I384N substitution does not alter the protein level of endogenous tubulins **(A)** Immunoblot analysis of HEK-293 cells transiently transfected with FLAG-tagged TUBA1A (WT and mutant) did not document any change in the protein levels of endogenous α and β tubulins. GAPDH was used as loading control. Histogram shows the quantitative analysis for western blot.The graph reports the mean ± SEM. Data were analyzed by Mann Whitney test. N=4. **(B)** Immunoblot analysis of COS-1 cells transiently transfected with FLAG-tagged TUBA1A (WT and mutant) did not document any change in the protein levels of endogenous α and β tubulins. GAPDH was used as loading control. Histogram shows the quantitative analysis for western blot.The graph reports the mean ± SEM. Data were analyzed by Mann Whitney test. N=4 (reproduced with permission from 'GeneDx '(1/31/23 )).


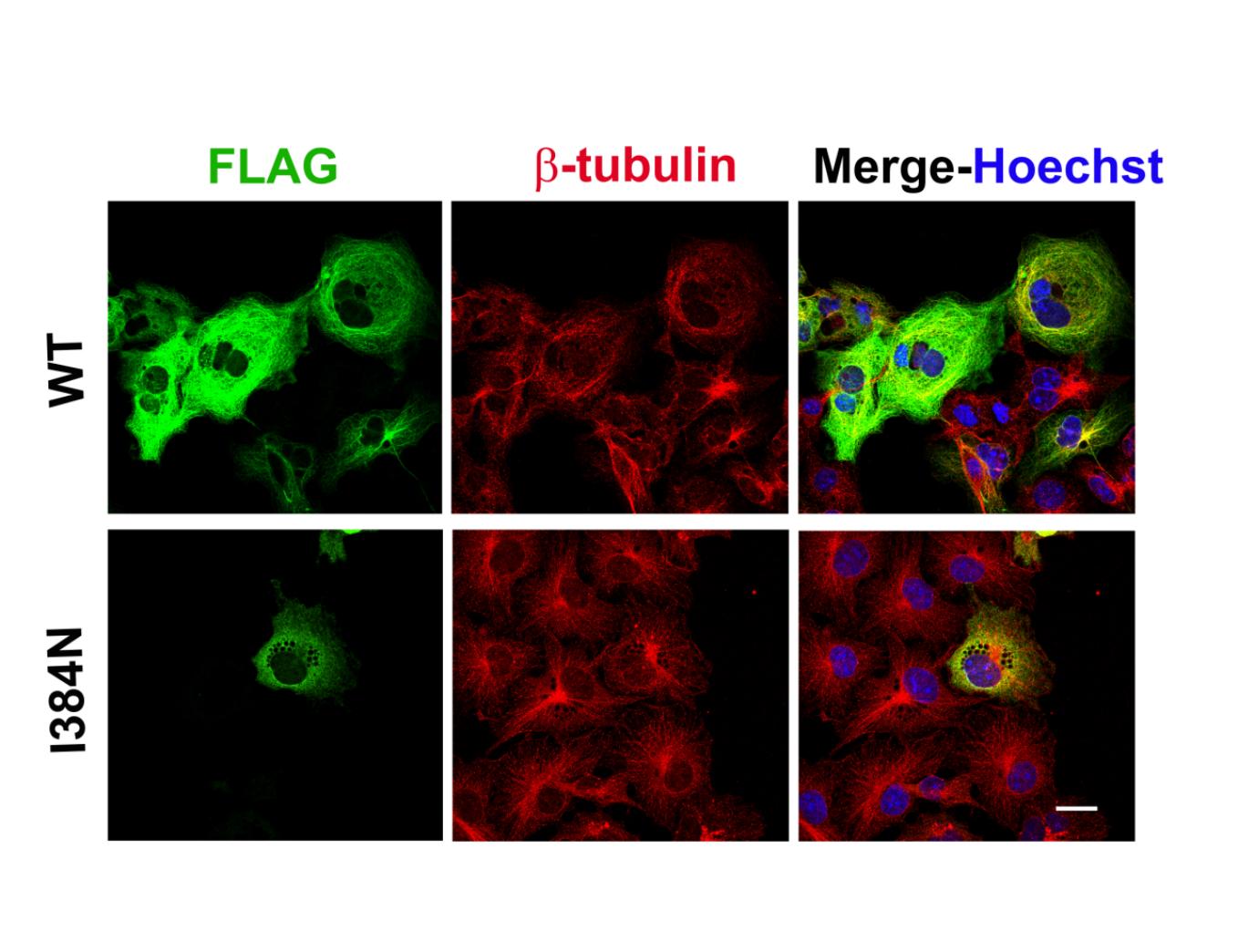


**Supplementary Figure 5.** The p.I384N substitution does not alter the structure of endogenous microtubules. Immunocytochemical analysis of COS-1 cells over-expressing FLAG-tagged TUBA1A (WT and mutant) showed that the p.I384N substitution does not impair the structure of endogenous microtubules, labeled for β-tubulin (red). WT and mutated TUBA1A were labeled for FLAG (green). Scale bar: 25 µm. (reproduced with permission from 'GeneDx '(1/31/23 )).


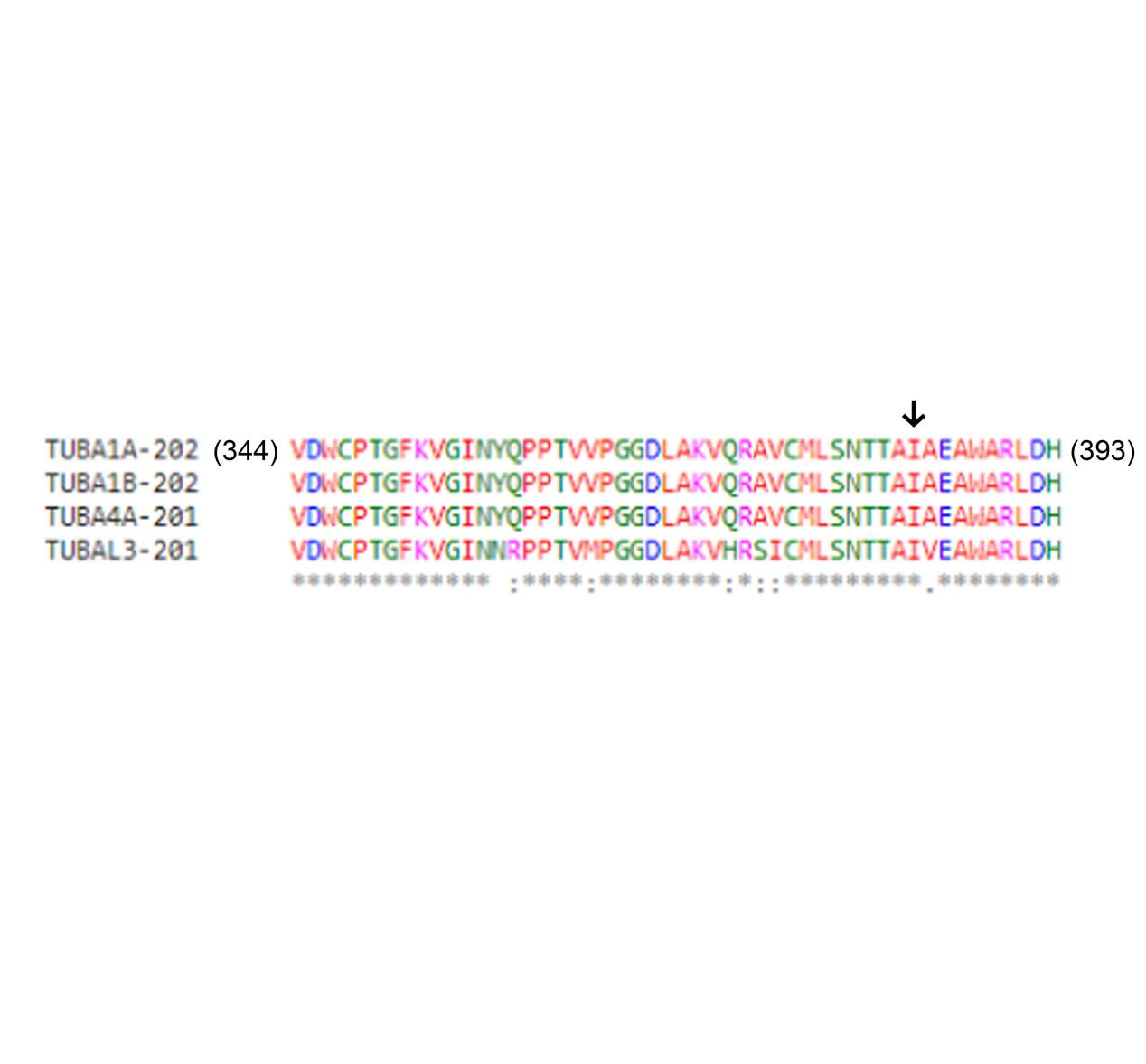


**Supplementary Figure 6.** The isoleucine in position 384 is highly conserved among α-tubulin paralogs. Multiple sequence alignment (ClustalW) of the different human α-tubulin isotypes around the isoleucine in position 384 (indicated by the arrow). “*” indicates perfect conservation ,“:” indicates strong similarity, “.” indicates weak similarity.


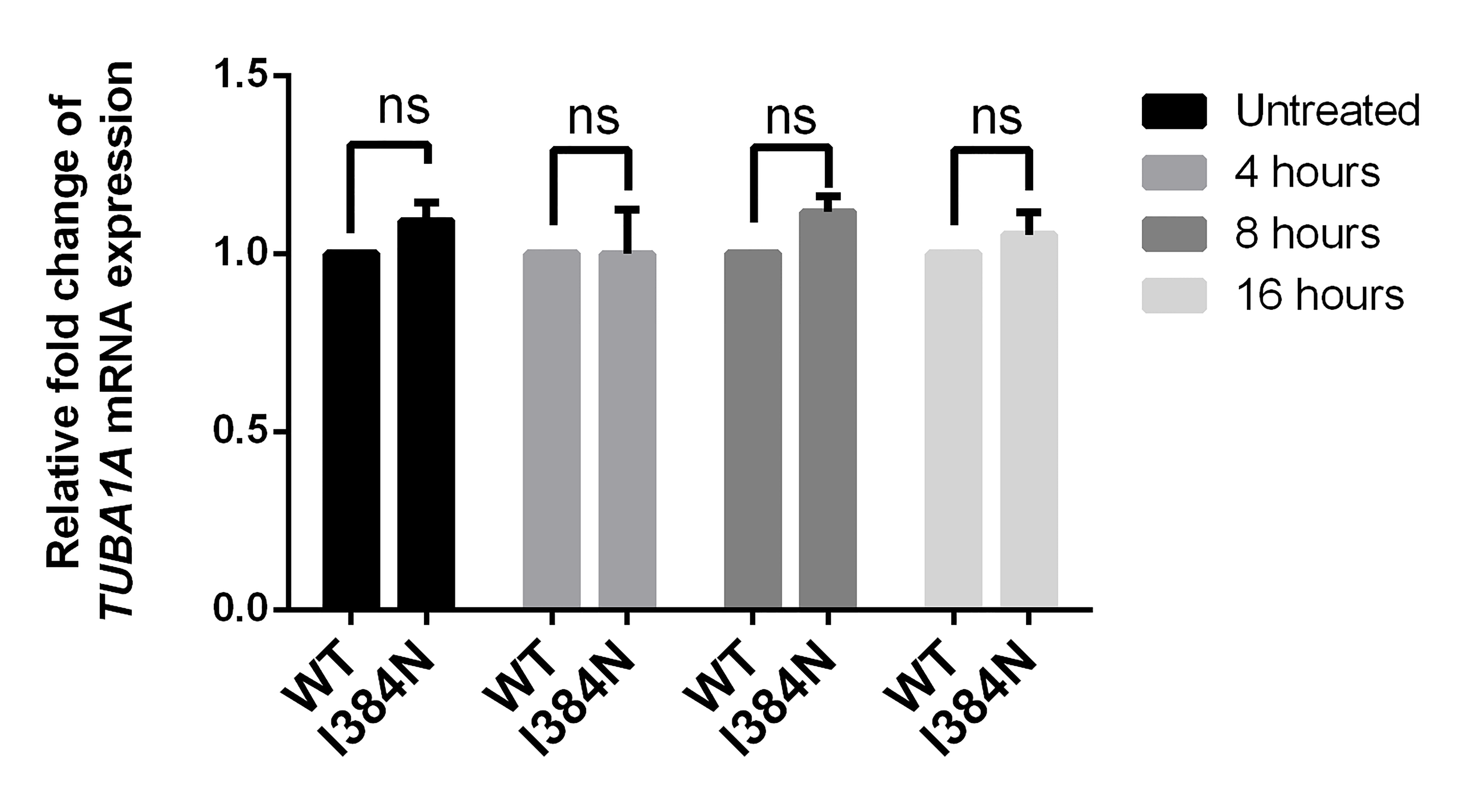


**Supplementary Figure** 7. Evaluation of *TUBA1A* mRNA expression in transiently transfected HEK-293 cells, untreated or treated with MG132. qRT-PCR experiments conducted on HEK-293 cells over-expressing WT and mutated TUBA1A recombinant proteins did not documented differences in *TUBA1A* mRNA levels. Expression values were normalized to *HPRT* expression. Hisogram shows the quantitative analysis of *TUBA1A* gene expression. The graph reports the mean ± SEM. Data were analyzed by Mann Whitney test. N =3


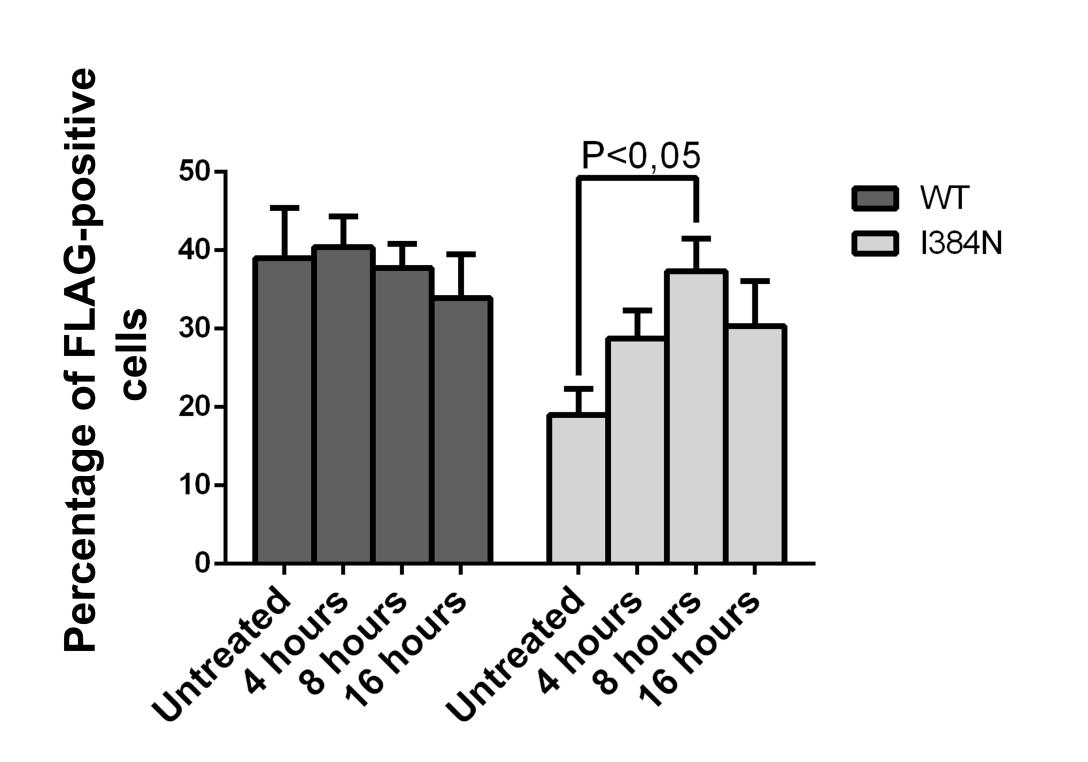


**Supplementary Figure 8.** Proteasome inhibition increases the percentage of FLAG-positive COS-1 cells over-expressing the mutated FLAG-tagged TUBA1A protein. The histogram shows the percentage of COS-1 cells over-expressing FLAG-tagged TUBA1A (WT and mutant) which were positive for the FLAG-staining, before and after different times (4, 8, 16 hours) of MG132 treatment. Transfection levels were calculated as: (Number of transfected cells/Number of total cell) x 100, of WT and mutated TUBA1A. The graph reports the mean ± SEM (587 cells from untreated WT, 362 cells from WT after 4 hours of MG132 treatment, 377 cells from WT after 8 hours of MG132 treatment, 338 cells from WT after 16 hours of MG132 treatment, 319 cells from untreated p.I384N, 340 cells from p.I384N after 4 hours of MG132 treatment, 310 cells from p.I384N after 8 hours of MG132 treatment, 328 cells from p.I384N after 16 hours of MG132 treatment). Data were analyzed by one way Anova. (reproduced with permission from 'GeneDx '(1/31/23 ).
